# Cancer: Save Our (Young) Skins!

**Published:** 2005-10

**Authors:** Victoria McGovern

Traditionally an adult disease, melanoma—the deadliest form of skin cancer—is on the rise in both children and adults around the world. In the United States, the overall rate of increase across the population was 2.8% per year between 1981 and 2001, according to data from the National Cancer Institute’s Survey of Epidemiology and End Results. People under age 20, a group in which melanoma is rare, have faced an overall 1.1% annual increase in disease incidence over the same period. But the rate among 10- to 24-year-olds has increased by 3.0%, according to research in the 20 July 2005 issue of the *Journal of Clinical Oncology*.

Julie Lange, an assistant professor of surgery and oncology at Johns Hopkins University School of Medicine in Baltimore, says, “Part of the apparent rise may be that cases ten or twenty years ago were not as likely to be reported to a tumor registry.” Reporting is more complete today, she says, and in some areas outpatient cases are now routinely reported along with inpatient cases. Improved reporting methods are not the whole story, though. “The incidence probably truly is increasing—it’s a fairly consistent finding,” Lange says.

Melanoma in children occurs so rarely that annual rate increases are measured in fractions of cases per million, so relatively small numbers of new cases can produce substantial percent changes in incidence. “From a public health burden point of view, saying it has increased from five cases to six cases per million children over a decade is more appropriate,” explains Ahmedin Jemal, the American Cancer Society’s program director for cancer occurrence.

The picture across the full human population is complex. “What we are seeing in adults, at least in Australia, is that amongst the older generation, their rates of melanoma are still climbing. We’re seeing the effects of their sun exposure fifty, sixty, seventy years ago,” says David Whiteman, a senior research fellow at the Queensland Institute of Medical Research in Brisbane, Australia. Increased attention to sun exposure seems to be working. “Amongst the younger [adult] cohort—the under-fifties and particularly the under-forties and younger—we’re seeing that their rates of melanoma are not as high as previous birth cohorts at the same age.”

Sun exposure and experience of blistering sunburns have been identified as important risk factors for adult melanoma. “Because we believe that UV exposure increases melanoma risk in adults, we are assuming that the same is true for children—whether there are other important factors for kids today, no one knows,” Lange says.

Whiteman’s group did a case–control study of childhood melanoma in Queensland in the 1990s to look for other such factors. “We were very interested in . . . exposure to pesticides, exposure to other chemicals, other environmental factors,” he says, “but we really found no differences in [those] exposures.” The group did find, however, that children with melanoma had more large noncancerous moles, heavier facial freckling, and less ability to tan compared to children without melanoma; they were also more likely to have a family history of the disease. These findings appeared in the January 1997 issue of the *International Journal of Cancer*.

Factors not yet investigated may also play a role. The Harvard Nurses’ Health Study, a long-term prospective study of risk factors for chronic diseases in women, has shown an association between orange juice consumption and melanoma in adult females. The investigators hypothesize that a photosensitizing compound in oranges may contribute to risk, says Diane Feskanich, an assistant professor of medicine at Harvard and an investigator on the study. However, a parallel study in men, not yet published, did not find the same strong association. “Whether there are photosensitizing foods is an open question,” she says. “Certainly there are drugs that warn you ‘don’t go out in the sun if you’re taking this.’”

Awareness of the risks of sun exposure has improved, according to Lange. “The population in general is more aware today of the potential danger from the sun than twenty or thirty or forty years ago,” she says. The same is true in Australia, which has among the world’s highest incidence of the disease. “The current generations of children are probably getting less sun exposure and fewer episodes of sunburn,” says Whiteman.

But better awareness of the major risks has not necessarily translated into complete protection of children. Even grasping the extent of older children’s exposure to the best-known risk factor, UV light, can be difficult. Despite prevention messages, many teenagers and young adults still want suntans. “The use of indoor tanning facilities is common among teenagers,” Lange says (in a 2003 survey, 47% of white girls aged 18 or 19 had used tanning beds three or more times). “Teenagers practice a lot of risky behaviors, and exposure to UV light is one of those behaviors.”

## Figures and Tables

**Figure:**
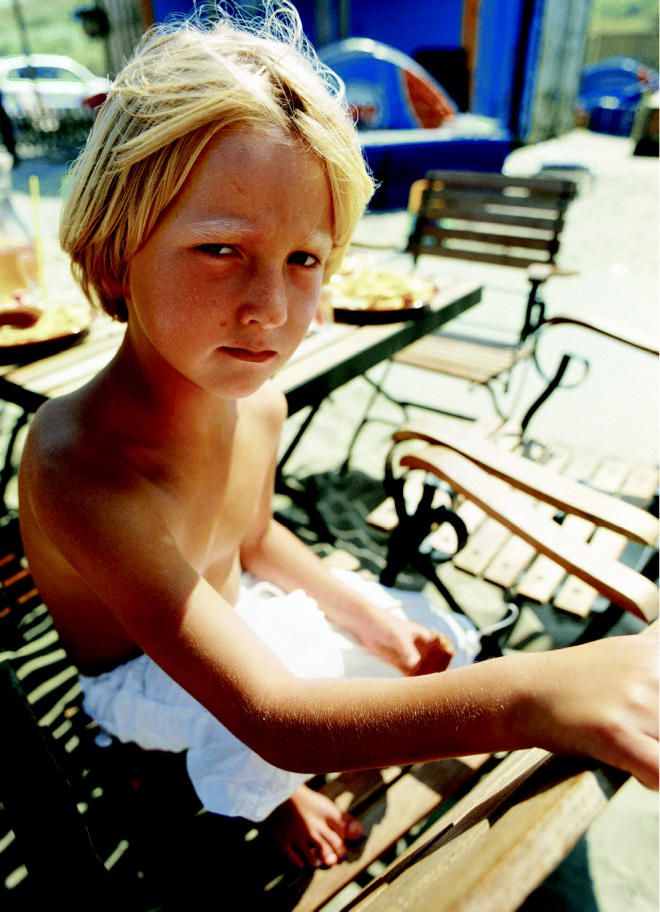
Burning youth. Despite better awareness of the risks of sun exposure, melanoma is climbing among young people, a heretofore largely unaffected group.

